# Agora: An openly available web resource to enable exploration of nascent therapeutic targets for Alzheimer’s disease

**DOI:** 10.1002/alz.089824

**Published:** 2025-01-03

**Authors:** Jessica S Britton, Jesse C Wiley, Jaclyn Beck, Karina Leal, Lawrence Yi, Brad Macdonald, Khai Do, Stockard Simon, Hallie Swan, Nicholas Grosenbacher, Thomas Yu, Jay Hodgson, Anna Greenwood

**Affiliations:** ^1^ Sage Bionetworks, Seattle, WA USA

## Abstract

**Background:**

Agora (https://agora.adknowledgeportal.org) is an openly available web resource developed to enable a broad spectrum of Alzheimer’s disease (AD) researchers access to target‐based evidence generated within the translational research portfolio of the National Institute on Aging (NIA). Agora aims to accelerate AD research and maximize therapeutic discovery by sharing information using interactive tools, data visualizations, and summarized evidence.

**Method:**

Agora enables the sharing of information about potential AD therapeutic targets by surfacing data visualizations, summary evidence, and results from targeted validation studies. Agora users can browse a list of over 950 potential therapeutic targets that have been nominated by the NIA’s Accelerating Medicines Partnership in AD (AMP‐AD) consortium and Target Enablement to Accelerate Therapy Development for AD (TREAT‐AD) centers, and by the broader AD research community; see visualizations and summarized information based on harmonized genome‐wide analyses of high‐dimensional human transcriptomic, proteomic, and metabolomic data; use interactive tools designed to enable non‐bioinformaticians to evaluate and compare complex multi‐omic data; and access the underlying data that drives the results and visualizations presented in the application.

**Result:**

Agora presents information and results about AD targets using approaches that make this information accessible to a broad spectrum of AD researchers. Recent updates to Agora include 1) comparative and per‐target visualizations for SRM proteomic differential expression results and 2) enabling the discovery of TREAT‐AD Target Experimental Resources for supported targets.

**Conclusion:**

The advancement of promising new AD therapeutics requires efforts from research groups and specialties across both academia and industry. Agora enables the AD research community to develop and share target hypotheses that will accelerate the investigation of promising new AD therapeutic targets, pathways and mechanisms.

